# On-site physiotherapy in older emergency department patients following a fall: a randomized controlled trial

**DOI:** 10.1007/s41999-024-01091-x

**Published:** 2024-11-16

**Authors:** Jonathan Benhamou, Tanguy Espejo, Henk B. Riedel, Thomas Dreher-Hummel, Ana García-Martínez, Barbara Gubler-Gut, Joris Kirchberger, Jan-Arie Overberg, Guido Perrot, Roland Bingisser, Christian H. Nickel

**Affiliations:** 1 Emergency Department, University Hospital Basel, Petersgraben 2, 4031 Basel, Switzerland; 2 Emergency Department, Hospital Clínic, C. de Villarroel 170, 08036 Barcelona, Spain; 3 Department of Therapies, University Hospital Basel, Spitalstrasse 21, 4031 Basel, Switzerland

**Keywords:** Randomized controlled trial, Physiotherapy, Falls, Older, Feasibility, Emergency department

## Abstract

**Purpose:**

Greater fear of falling (FOF) is associated with an increased risk of falling in patients aged 65 and older. This study aims to assess the impact of physiotherapy on FOF in older patients and investigates the feasibility of such an intervention in the emergency department (ED) setting.

**Methods:**

All patients aged 65 or older, who presented to the ED of the University Hospital Basel after a fall between January 2022 and June 2023 were screened for inclusion. Participants were assigned to an intervention or control group depending on the randomized presence or absence of a physiotherapist at inclusion. Both groups received the same fall prevention booklet. Physiotherapists instructed and performed exercises with patients in the intervention group. The primary outcome was the difference in FOF between groups 7 days post inclusion, assessed by short Falls Efficacy Scale International (sFES-I). Secondary outcomes included feasibility, overall reduction of FOF, patient satisfaction, the occurrence of falls post inclusion and the use of medical resources.

**Results:**

Of the 1204 patients screened for inclusion, 104 older adults with a recent fall were enrolled (intervention: n = 44, control: n = 60); median age was 81 years and 59.1% were female. There was no between-group difference in FOF as measured by sFES-I within a week of inclusion (p = 0.663, effect size = 0.012 [95% confidence interval (CI) – 0.377 to 0.593]). Despite the intervention being deemed feasible from the physiotherapist’s perspective, the study encountered challenges, such as low recruitment (with the planned sample size not being reached) and a notable dropout rate before the first follow-up.

**Conclusion:**

A physiotherapy intervention in the ED showed no improvement in FOF when compared to a control group.

**Trial registration:**

Trial registration number and date NCT05156944, 01.12.2021.

## Key summary points

**Aim:**

This study aims to assess the impact of physiotherapy on fear of falling (FOF) following a fall in older patients and investigates the feasibility of such an intervention in an emergency department (ED) setting.

**Findings:**

While having substantial recruitment issues, this study demonstrated that physiotherapy in the ED did not improve FOF in older adults who had presented with a fall compared to a control group.

**Message:**

Factors that might explain the lack of impact of our intervention on FOF are patient selection (included patients had moderate FOF and low anxiety at inclusion), the intervention (timing, frequency, and duration) and the outcome measure (as measuring avoidance behavior associated with FOF might have been more clinically meaningful).

## Introduction

### Background and objectives

Falls are among the most frequent reasons for presentation to an emergency department (ED) in patients aged 65 years and older [1]. Approximately one-third of community-dwelling older adults and nearly one half of institutionalized individuals suffer from a fall each year [2, 3]. Falls are associated with injuries, functional decline, social withdrawal, anxiety, depression and an increased use of medical resources [4]. While prevention of falls and ED revisits may be relevant goals [5–8], other, more patient-centered outcomes, such as a reduced fear of falling (FOF), have been identified as research priorities [9]. FOF is common among older adults with falls, and has been associated with reduced physical activity, impaired mobility, decreased performance in activities of daily living, increased rates of institutionalization, and an increased risk of falling [10–15]. Furthermore, FOF in older adults presenting to the ED after a fall was linked to poor outcomes (including death, hospitalization and institutionalization) at long-term follow-up [16].

The world guidelines for fall prevention and management strongly (Grade 1B) recommend evaluating for FOF using a standardized instrument such as the Falls Efficacy Scale International (FES-I) or the short FES-I, with a positive screening warranting further risk stratification with gait and balance testing [17]. In addition, the world guidelines for fall prevention and management recommend early referral to physiotherapy for patients at an intermediate or high risk of falling, without providing data for the ED setting [17]. Several studies have suggested that the implementation of physical therapy could be considered feasible in an ED setting [18, 19]. ED-based physical therapy has been associated with a decrease in hospital admissions and fall-related ED revisits [5, 7, 8]. However, there is no randomized controlled trial (RCT) published investigating the impact of physiotherapy on FOF in an ED setting.

The aim of this RCT was to assess the effect of physiotherapy on FOF in older patients that presented to the ED with a fall within the past 7 days. In addition, we investigated the feasibility of the intervention, objective functional levels for the intervention group at day 7 and patients’ satisfaction with their ED work-up. Furthermore, we measured the course of FOF, occurrence of falls and utilization of medical resources throughout the 6-week study period.

## Methods

### Trial design

Our trial was designed as a monocentric, block-randomized, controlled, parallel-group trial. The study protocol was approved by the local Ethics Committee project N° 2021-02165, and the study is registered on the clinicaltrials.gov website (study N° NCT05156944). All patients gave written informed consent before inclusion. Data are presented according to the Consolidated Standards of Reporting Trials Guidelines [20].

### Participants

Patients aged 65 years and older who presented to the ED of the University Hospital Basel between January 2022 and June 2023 and had experienced at least one fall within 7 days of ED presentation were screened for inclusion. A fall was defined as “an event which results in a person coming to rest inadvertently on the ground or floor or other lower level and other than as a consequence of the following: sustaining a violent blow, loss of consciousness, sudden onset of paralysis, or an epileptic seizure” [21, 22]. Exclusion criteria were: admission to hospital after ED work-up (as these patients most likely benefit from physiotherapy during hospital stay), immobilizing fractures of the lower extremities, inability or contraindications to undergo the intervention or follow the study procedures e.g. due to neurological disorders (such as hemiplegia), non-Swiss German-speaking, inability to follow instructions or to provide consent due to cognitive impairment, and prior enrolment in this trial. Patients were recruited during weekdays from 8 a.m. to 5 p.m. due to the presence of the study physicians.

### Interventions

 At presentation, a detailed falls and medical history was taken, and a clinical examination was performed by the ED physician in charge. Where indicated, a further assessment for underlying causes, e.g., an electrocardiogram and an orthostatic blood pressure were conducted. Blood tests and screening for cognitive impairment [23, 24] were conducted where deemed necessary. A depression and anxiety screening (using the Hospital Anxiety and Depression Scale (HADS), which is a validated screening tool consisting of 14 questions with 7 each for depression and anxiety [25, 26]) was performed on all patients, as was an assessment for frailty using the Clinical Frailty Scale (CFS) [27–29]. An evaluation of FOF was done using the sFES-I (which includes questions regarding everyday activities such as getting dressed or undressed, taking a bath or shower, getting in or out of a chair, going up- or down-stairs, reaching for something overhead or on the ground, walking up or down a slope, and going out to a social event) [30].

### Intervention group

The intervention in the ED, performed by a physiotherapist (PT), consisted of a physiotherapeutic assessment, the short physical performance battery (SPPB) [31], information on the expected course of the condition and instructions on self-management (e.g., staying active, adaptation of behavior and surroundings at home). Additionally, as recommended in the world guidelines for falls prevention and management [17], a strength exercise (sit-to-stand) and a balance exercise consisting of three difficulty levels (1. Stand behind a chair, feet shoulder-width apart. Try to let go of the chair for 30 sec; 2. Same as exercise 1 but with feet together; 3. Same as exercise 2 but in a tandem stance) for daily, self-guided therapy were instructed and performed with the patients. The PT recommended the patients do these exercises at least five times a day, while trying to incorporate them into daily routine activities. In addition, a fall prevention booklet issued by the Swiss Council for Accident Prevention with an activity plan, an exercise description, a checklist on fall hazards at home and fall prevention behavior was handed out [32]. The intervention was conducted at the bedside in the ED examination room and took an average of 23 min.

Over the course of the study, two different PT administered the intervention, one for the first 7 months and the second for the remaining 11 months. Both PTs adhered strictly to the study program. Fall prevention is an integral part of the curriculum of studies in physical therapy, with both PTs being equally qualified.

### Control group

Patients in the control group were given the same booklet as the intervention group by study physicians, but without an instruction by a PT and without performing the exercises in the ED. No SPPB was performed as no PT was present at inclusion for this group. The control group “intervention” took an average of 2 min.

### Follow-up

At the first follow-up on day 7, patients in both the control and intervention group received a scheduled, in person consultation by a PT, either at home or at the study site. During this visit, patients were asked for the number of exercises performed and which recommended interventions had been implemented. In addition, objective functional levels were assessed using SPPB, and patients were instructed to answer questionnaires concerning their FOF (sFES-I) and the use of medical resources since inclusion. The follow-ups on day 21 and day 42 consisted of telephone interviews conducted by 2 study physicians (TE, HBR) using the same questionnaires as on days 0 and 7.

### Outcomes

The primary outcome was the difference in FOF between groups at day 7, assessed by sFES-I. The sFES-I was validated and shown to have excellent reliability and construct validity (Cronbach’s alpha 0.92) [30]. Cut-offs were defined to differentiate between low, moderate, and high FOF (7–8, 9–13 and 14–28) [33].

Secondary outcomes were the feasibility of the PT intervention as assessed by eligibility, recruitment, loss to follow-up, dropout rates, and a questionnaire (designed by the authors of the study to best assess feasibility from a PT point of view) filled out on the day of inclusion by a PT. Additional outcomes were reduction of FOF over the course of the whole study, objective functional levels in the intervention group, as measured by SPPB at day 7, patients’ satisfaction with their ED work-up (which was assessed using a questionnaire on day 7), the occurrence of falls following randomization from patient recollection, and the use of medical resources.

### Sample size

For the sample size calculation, we considered studies using the FES-I and sFES-I to derive the estimated benefit of our intervention [34–37]. We calculated a necessary population of 64 patients per group to detect a mean difference of three points (power = 80%, *α* = 0.05) with an effect size of 0.5. Based on expert consensus (TDH, AGM, BGG, GP, RB, CHN), we defined a priori that a mean difference of three points could be deemed clinically meaningful. Assuming a 10% drop out rate or secondary exclusion due to hospitalization, our study population was set at 70 patients per group, which is 140 patients total.

### Randomization

A block randomization was performed. A PT was present in the ED for 50% of the inclusion timeframes (randomized by REDCap), and patients were assigned to groups depending on the presence or absence of a PT. Eligible patients were approached by the study physicians, who were not blinded. Patient blinding was achieved by giving patients written informed consent in the ED without informing them on their group allocation and the exact aims of the study. At the first follow-up on day 7, patients were informed on group allocation.

### Statistical methods

Missing data were handled with available case analysis. Due to a high dropout rate (especially in the control group) with the risk of attrition bias and potential contamination bias due to crossover, a per protocol analysis was conducted. The data were tested for normal distribution; a Student’s *t* test was used for normally distributed data and a Wilcoxon test for non-normally distributed data. The level of significance was two-sided, with a significance level of *α* = 0.05. The results were corrected for the confounding factors age, sex, and frailty (measured by CFS) using a logistic regression model. All analyses were done using R (Version 4.3.1).

## Results

### Recruitment

**Fig. 1:**
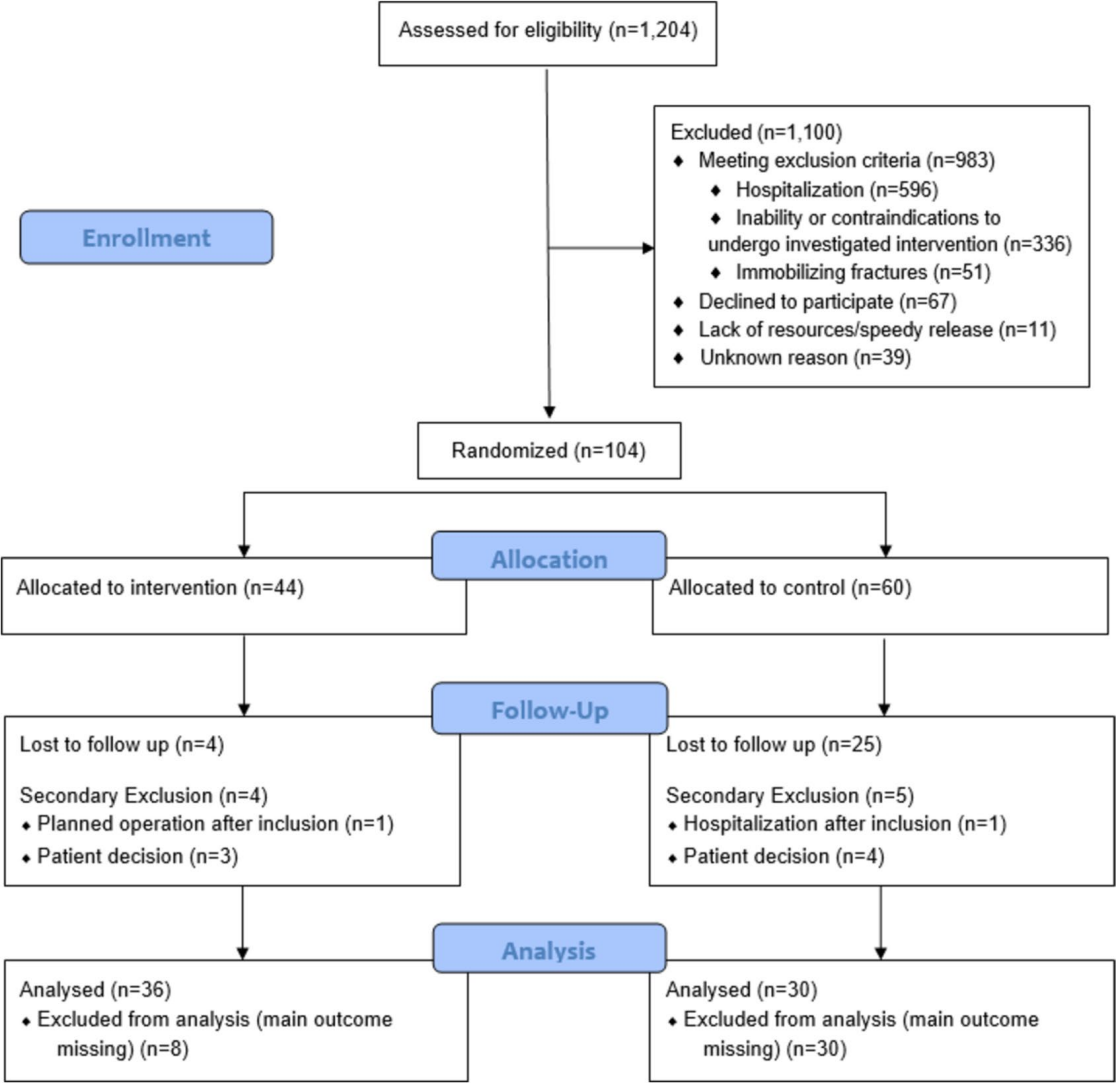
Consolidated Standards of Reporting Trials (CONSORT) flow diagram of trial participants

Of the 1204 patients screened between January 2022 and June 2023, 1100 patients were excluded (Fig. 1). Nine hundred and eighty three (89.4%) of the exclusions were due to hospital admission or contraindications to undergo the investigated intervention. The remaining 104 patients were assigned to intervention and control groups based on the randomized presence or absence of a PT at presentation. Of the 60 patients allocated to the control group, 30 dropped out before the first follow-up (25 were lost to follow-up; 4 patients withdrew their consent, and 1 patient was excluded in a secondary step due to unforeseen hospitalization). Of the 44 patients in the intervention group, 4 were lost to follow-up before the first follow-up, 3 patients withdrew their consent, and 1 patient withdrew due to an intercurrent operation after inclusion. The remaining 30 patients in the control group and 36 patients in the intervention group were analyzed. There were no differences in baseline characteristics between the per protocol and dropout populations. A further four patients per group were lost to follow-up at the final follow-up (day 42), leaving 26 patients in the control group and 32 in the intervention group who completed the study as per protocol.

### Baseline data

An overview of the baseline characteristics is shown in Table 1. Median age was 81 years (IQR 77–88) in both groups and 57.7% were female. There was no between-group difference at inclusion for triage level as measured by the Emergency Severity Index (ESI) (*p* = 0.842), frailty level as measured by CFS (*p* = 0.324), number of falls in the past 12 months (*p* = 0.259), mobility (*p* = 0.349), or pain as measured by numeric rating scale (*p* = 0.664). With median sFES-I scores of 9.0 in the control group and 10.0 in the intervention group, both groups were in the “moderate concern of falling” category (*p* = 0.03). Overall, 25.0% of patients took pain medication before inclusion. In the HADS, both groups had median scores within the normal range (0–7 points), indicating no clinically significant depression or anxiety [26].

**Table 1:** Baseline characteristics at inclusion

	Overall (*N* = 104)	Control (*N* = 60)	Intervention (*N* = 44)
Age in years, median (IQR)	81 (77–88)	81 (76–87)	81 (76–88)
Female sex, *n* (%)	60 (57.7)	36 (60.0)	24 (54.5)
ESI, *n* (%)			
1	3 (2.9)	1 (1.7)	2 (4.5)
2	10 (9.6)	6 (10.0)	4 (9.1)
3	80 (76.9)	47 (78.3)	33 (75.0)
4	11 (10.6)	6 (10.0)	5 (11.4)
CFS, *n* (%)			
1	1 (1.0)	0 (0)	1 (2.3)
2	9 (8.6)	5 (8.3)	4 (9.1)
3	36 (34.6)	23 (38.3)	13 (29.5)
4	21 (20.2)	9 (15.0)	12 (27.3)
5	14 (13.5)	7 (11.8)	7 (15.9)
6	15 (14.4)	11 (18.3)	4 (9.1)
Missing	8 (7.7)	5 (8.3)	3 (6.8)
BMI (kg/m^2^), median (IQR)	24 (22–28)	24 (21–28)	25 (22–27)
Pain medication before inclusion, *n* (%)	26 (25.0)	18 (30.0)	8 (18.2)
NRS Pain Score, median (IQR)	2.0 (0–5.0)	2.0 (0–5.0)	1.5 (0–5.0)
Mobility, *n* (%)			
Independent	72 (69.2)	36 (60.0)	36 (81.8)
Use of a cane or walking stick	14 (13.5)	11 (18.3)	3 (6.8)
Use of two canes or a walker	18 (17.3)	13 (21.7)	5 (11.4)
Weekly physical activity (minutes), median (IQR)	180 (60–360)	120 (30–360)	210 (68–420)
Additional fall in the past 12 months, *n* (%)	44 (42.3)	28 (46.7)	16 (36.4)
Number of falls in the past 12 months, median (IQR)	0 (0–1.0)	0 (0–1.0)	0 (0–1.0)
HADS Anxiety (day 0), median (IQR)	2.0 (1.0–4.0)	1.0 (0.0–2.0)	3.0 (2.0–5.0)
HADS Depression (day 0), median (IQR)	3.0 (1.0–5.0)	2.0 (1.0–4.0)	4.0 (2.0–6.3)
Short FES-I (day 0), median (IQR)	9.0 (8.0–11)	9.0 (7.0–10)	10 (8.0–12)

*IQR* interquartile range, *ESI* emergency severity index, *CFS* clinical frailty score, *BMI* body mass index, *NRS* numeric rating scale, *HADS* hospital anxiety and depression scale, *FES-I* falls efficacy scale – international

### Primary outcome

**Fig. 2:**
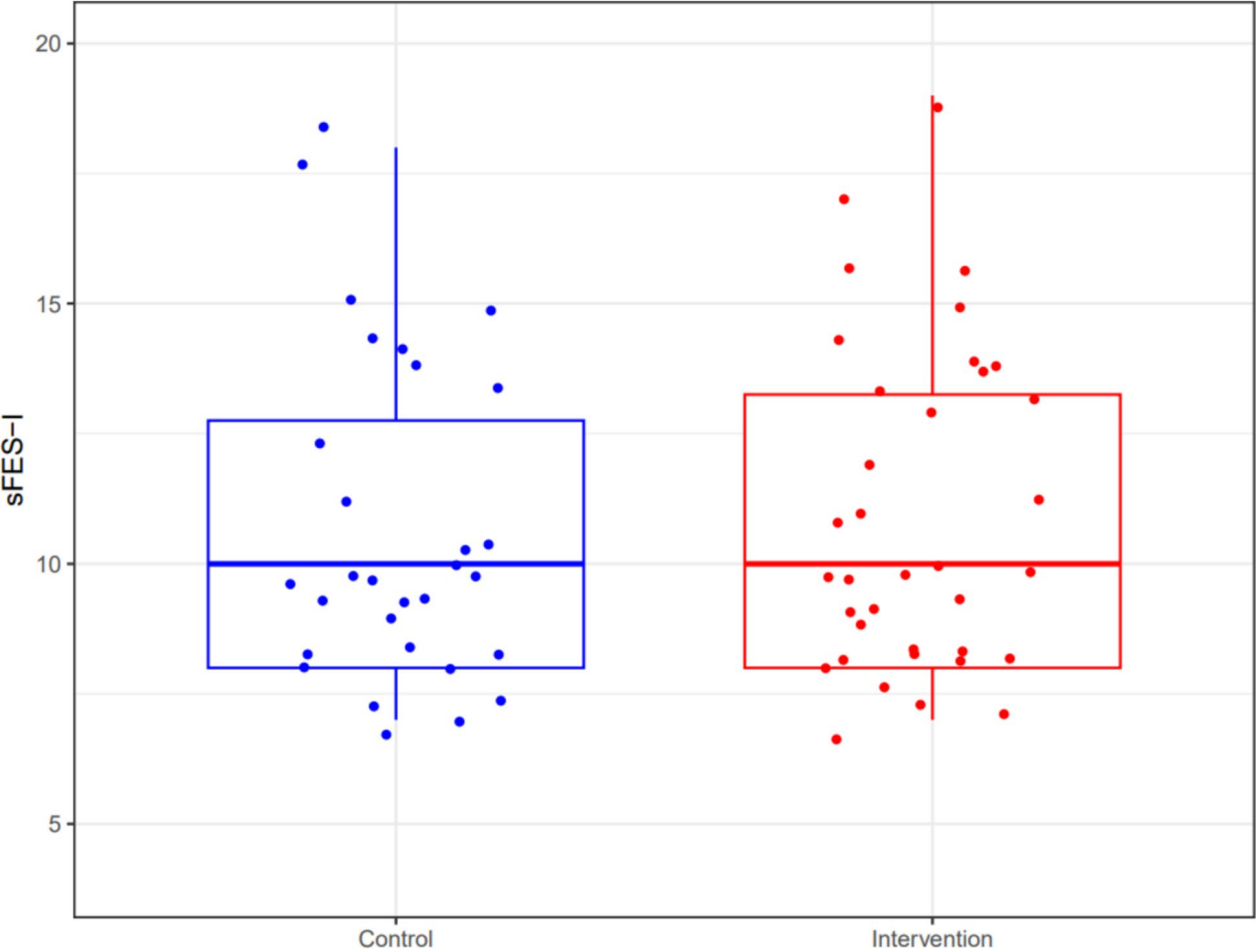
Boxplot comparing short Falls Efficacy Scale International scores (7 to 28 points) between the intervention and control group at day 7 after inclusion

The median sFES-I on day 7 was 10 (IQR 8–13) in both the intervention and control group (*p* = 0.663, effect size = 0.012 [95% confidence interval (CI)—0.377 to 0.593]) (see Table 2 and Fig. 2).

**Table 2:** Outcomes at day 7 after inclusion

	Overall (*N* = 66)	Control (*N* = 30)	Intervention (*N* = 36)	*P* value
Drop out/Loss to follow-up, *n* (%)	38 (36.5)	30 (50.0)	8 (18.2)	
Death, *n* (%)	0 (0.0)	0 (0.0)	0 (0.0)	
Location of follow-up, *n* (%)				0.886
Hospital	26 (39.4)	11 (36.7)	15 (41.7)	
Home	38 (57.6)	18 (60.0)	20 (55.5)	
Missing	2 (3.0)	1 (3.3)	1 (2.8)	
Falls since inclusion, *n* (%)	2 (3.0)	2 (6.7)	0 (0)	0.394
Pain medication since inclusion, *n* (%)				0.628
No	43 (65.2)	21 (70.0)	22 (61.1)	
Yes	22 (33.3)	9 (30.0)	13 (36.1)	
Missing	1 (1.5)	0 (0.0)	1 (2.8)	
NRS Pain Score, median (IQR)	2.0 (0–5.0)	0 (0–5.0)	3.0 (0–5.8)	0.109
Short FES-I (day 7), median (IQR)	10 (8.0–13)	10 (8.0–13)	10 (8.0–13)	0.663
SPPB (day 7), median (IQR)	8.0 (6.0–10)	8.0 (4.3–10)	8.0 (7.0–9.0)	0.636

*IQR* interquartile range, *NRS* numeric rating scale, *FES-I* falls efficacy scale – international, *SPPB* short physical performance battery

### Secondary outcomes

The results of the feasibility questionnaire filled out by the PT are seen in Table 3.

**Table 3:** Feasibility of the intervention on day of inclusion from the physiotherapist’s point of view

	Intervention (*N*=36)
Missing values, *n* (%)	2 (5.6)
Number of interruptions during the intervention, *n* (%)	
0	17 (47.2)
1	14 (38.9)
2	2 (5.6)
3	1 (2.7)
Could the patient’s medical needs be met using the standardized physical therapy approach?*, median (IQR)	5.0 (5.0–6.0)
Could the patient’s psychosocial needs be met using the standardized physiotherapy approach?*, median (IQR)	5.0 (5.0–6.0)
Was the patient open to a physiotherapeutic intervention in the ED?*, median (IQR)	6.0 (5.0–6.0)
Did disturbances in the organizational integration (planning patients, interruptions, transfers within the emergency center, noise, or chaos) of the physiotherapy intervention in the emergency center occur from the perspective of physiotherapy?*, median (IQR)	1.0 (1.0–2.0)
Does a physiotherapeutic intervention in this case at this point in time make sense from a physiotherapeutic perspective?*, median (IQR)	5.0 (4.0–6.0)
Did the interprofessional collaboration from the physiotherapy perspective regarding the exchange of information work?*, median (IQR)	5.5 (5.0–6.0)

*IQR* interquartile range

*On a scale of 1 (= no) to 6 (= yes)

Over the course of the study, the median sFES-I improved in both groups. On the day of inclusion, the median sFES-I was 9 (IQR 7–10) in the control group and 10 (IQR 8–12) in the intervention group. At the last follow-up on day 42, the median sFES-I was 7 (IQR 7–9) in the control group (*p* < 0.001, effect size = 1.335 [95% CI 0.969 to 1.700]) and 8 (IQR 7–9.3) in the intervention group (*p* < 0.001, effect size = 1.065 [95% CI 0.638 to 1.492]).

**Fig. 3:**
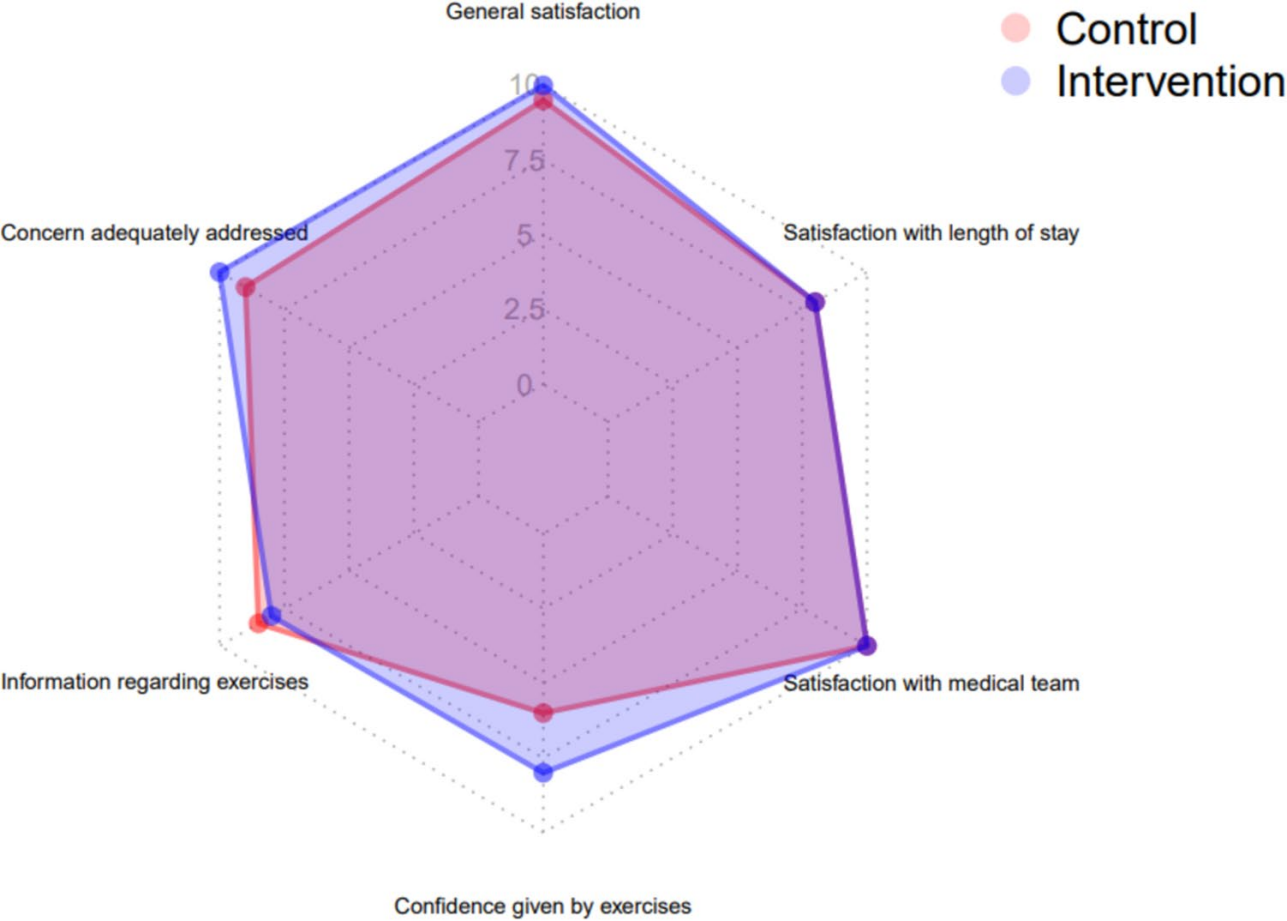
Radar chart of patient satisfaction with ED work-up (assessed on day 7 after inclusion)

The mean SPPB measured in the intervention group at baseline was 6.14. The mean SPPB for the intervention group on day 7 was 7.97 (*p* = 0.001, effect size = − 0.749 [95% CI − 1.197 to − 0.302]) (Table 2). There was no difference in patient satisfaction with their ED work-up (Fig. 3).

At the follow-up on day 7, 2 patients in the control group reported a fall (one each) while no patient reported a fall in the intervention group (*p* = 0.394). At the follow-up on day 21, 1 additional patient reported a fall in each group (*p* = 1.000). At the follow-up on day 42, 4 additional patients reported a fall in the control group and 2 additional patients reported a fall in the intervention group (*p* = 0.482).

Concerning the use of medical resources, there was no between-group difference at each of the three follow-ups for ED visits since inclusion, visits to a general practitioner (GP) since inclusion, hospitalization since inclusion, physiotherapy since inclusion or imaging since inclusion (Table 4).

A multivariable regression analysis for confounding factors age, sex and frailty measured by CFS before inclusion showed that only age had a statistically significant impact on sFES-I score (*p* = 0.025) at day 7.

During the first follow-up, adherence to the recommendations (environmental interventions as well as balance and strength exercises) was assessed in both groups by the PT. A higher number of patients in the intervention group followed the recommendations in all cases (Table 5). After removal of non-adherent patients, the median sFES-I was 9 (IQR 8–10) in the control group and 10 (IQR 8–13) in the intervention (*p* = 0.264, effect size = 0.127 [95% CI − 0.238 to 0.947]).

### Harms

We observed one serious adverse event in the control group and none in the intervention group. Serious adverse events were defined as medical occurrences that either resulted in death or were life-threatening, required in-patient hospitalization, or resulted in persistent or significant disability or incapacity. In our case, the patient had another injurious fall that required hospitalization.

## Discussion

### Interpretation

In our RCT of 104 older ED patients presenting after a fall, no difference in FOF was observed between a physiotherapeutic intervention group and a control group 7 days after inclusion.

Existing literature presents mixed findings on the impact of physiotherapy on FOF. A previous RCT indicated a 10% reduction in FOF through a lay-led home-based program including physical training over 12 weeks, but the participants had higher baseline FOF scores, received frequent visits from trained volunteers, and the controls had no instructions at all [36]. A Canadian study reported FOF improvement in community-dwelling older adults after a multifactorial intervention over 6 weeks, supervised by a PT and volunteers, but lacked a control group [38]. Conversely, a Dutch geriatric rehabilitation study reported no reduction in FOF after a multi-component intervention integrated in physical therapy sessions for patients following a hip fracture [39]. In all aforementioned studies, the intervention included guided physical therapy over several weeks, as opposed to the single session patients received in the intervention group in our study. Therefore, timing, frequency, and duration of the intervention might have been a reason for the lack of impact on FOF.

A mean difference of three points in the sFES-I may have been too high, especially given that our patients had moderate FOF at baseline. Furthermore, FOF alone might not have been the right primary outcome. A qualitative study in people with FOF found that while certain individuals were not affected by their FOF, others developed avoidance of certain activities [40]. Additionally, patients with disproportionately high FOF were more likely to develop avoidance behavior [41, 42]. Such avoidance behavior has been associated with poor physical performance, limitations in activities of daily living, and disability [15, 43]. A questionnaire measuring avoidance behavior due to FOF, such as the FES-IAB (in which AB stands for avoidance behavior) [44], may have provided deeper insight into the clinical meaningfulness of the measured FOF.

Another reason for a lack of impact on FOF might have been patient selection. A prospective cohort study demonstrated that 60% of community-dwelling older adults had persistent FOF [45], and FOF has been shown to decline linearly over time, becoming negligible 2 to 3.5 years after the last fall [46]. While it is conceivable that a certain degree of FOF following a fall may be normal, it is important to identify patients who would most benefit from interventions to reduce FOF. Anxious people, for example, might overestimate their risk of falling (even when the physiological risk is low) and subsequently develop avoidance behavior [42]. When assessing for FOF in the ED, it may be beneficial to also assess for anxiety and to monitor the course of FOF in patients with high scores more closely [47]. Patients in our study had moderate FOF and low scores for anxiety at inclusion and might therefore have benefitted less from the intervention.

While PT deemed the intervention feasible in our ED setting, a low recruitment and high dropout rate raise questions about the feasibility of such an intervention. In a study assessing ED-based PT and case management services following falls in older adults, only 24.3% of patients were admitted to hospital [7], as opposed to the 54.2% that were admitted over the course of our study. Several studies have tested the feasibility of fall prevention programs and the implementation of physical therapy in an ED setting with recruitment being the main challenge (recruitment rates of 11–67% of all eligible patients), even though adherence to recommendations remained high in all studies (88–100%) [18, 19, 48, 49]. In our cohort, the emergency physicians in charge may have been overly cautious regarding hospitalization.

Patients in the intervention group showed an improvement in objective functional levels, as measured by SPPB, 7 days post-inclusion. However, as no PT-performed measurements of SPPB at baseline for patients in the control group were available, it is unknown whether the improvement in SPPB can be attributed to the intervention.

In contrast to a recent RCT indicating a significant reduction in fall-related ED revisits with ED-based physiotherapy [5], our study found no differences in ED revisits, GP visits, hospitalizations, number of physiotherapy sessions, or imaging between intervention and control groups. Potential differences to the aforementioned study may be associated with the relatively good health of our patients (74% using assistive devices in the referenced study, compared to 22.7% in our trial). In addition, admission to hospital was not an exclusion criterion in that study (18.8% of patients were admitted after inclusion).

While adherence to recommendations was higher in the intervention group in our study, there is no direct evidence linking adherence to intervention efficacy and fall prevention [50].

In summary, while the PT intervention did not influence FOF, it did improve adherence to recommendations. Our findings should not fuel “therapeutic nihilism” for falls prevention or hinder future ED-based falls interventions. RCTs favor quantifying efficacy, promoting research in ideal conditions, often inconsistent with actual clinical practice [51]. In our case, ideal “standard-of-care” conditions were granted to both groups, as all patients were provided with a fall prevention booklet with exercises for daily self-guided therapy. This might represent a higher standard of care than usually provided. The notable sFES-I improvement observed in both groups at 6 weeks post inclusion, with no between-group difference, further supports the conclusion that FOF was not influenced by the PT intervention. Over the course of the study, the percentage of patients who attended physiotherapy sessions increased in both groups (20–60% in the control group, 22.2–58.3% in the intervention group), which might explain the reduction in FOF in both groups without between-group differences. Having a similarly high standard of care may benefit all older adults presenting to the ED following a fall.

### Limitations

The main limitation of our study was under-recruitment in all patients and a high dropout rate of participants in the control group before the follow-up visit at day 7, despite the intervention being deemed feasible by PT and a high on-site presence of the study physicians (who did not have clinical duties). A cautious hospitalization policy (which may have introduced sampling bias towards more healthy and less frail patients) and stringent exclusion criteria are possible explanations. Given the moderate FOF at baseline, we hypothesize that patients in the control group might have been less motivated to further participate in the study. This could have led to the significantly higher dropout rate in that group, introducing the risk for attrition bias (even though the dropout population did not differ significantly from those who completed the study). Furthermore, due to limited funding, recruitment had to be stopped before reaching the calculated group size of 70 each, leaving the trial underpowered and increasing the risk of a Type II error.

Moreover, an increase in physiotherapy visits in both groups over the course of the study suggests potential contamination bias from physiotherapy referrals by GPs. Possible unmeasured confounders that may have influenced our results include medications that increase fall risk, fall etiology and the number of previous injurious falls. In addition, recall bias may have limited the quality of data about falls before and after randomization [52].

### Generalizability

The generalizability of our findings may be limited by the fact that this study was conducted in a Swiss ED in a tertiary care center with a study population mainly consisting of patients of European descent. In addition, Switzerland has a well-funded healthcare system, and we speculate that the Swiss population may have very high health expectations and high health literacy, and therefore a low threshold for presentation, as well as a low threshold regarding access to medical resources, such as physiotherapy.

The small effect size in our intervention can be used for planning future studies. However, a recalculation of sample size based on this effect size showed that thousands of patients per group would have to be included for a significant difference between the intervention and the admittedly high standard of care. However, our sample size of 104 patients is sufficient for a pilot study of exploratory character [53].

### Conclusion

An individually adjusted physiotherapy intervention in the ED showed no improvement in short-term FOF as measured by sFES-I when compared to a control group. Several factors might explain the lack of impact of our intervention on FOF, such as patient selection (included patients had moderate FOF and low anxiety at inclusion), the intervention (timing, frequency, and duration) and the outcome measure (measuring avoidance behavior associated with FOF might have been more clinically meaningful). The high standard of care and the high use of physiotherapy after ED presentation might have led to a reduction in FOF in both groups over the course of the study.

## Data Availability

The dataset is not available due to ethical restrictions. Upon request, the release of the dataset can be requested from the responsible ethics committee.

## Appendix

See Tables 4 and 5.

**Table 4:** Utilization of medical resources (assessed on day 7, 28 and 42 after inclusion)

	Control (*N* = 30)	Intervention (*N* = 36)	*P* value
Follow-up day 7			
Physiotherapy since inclusion, *n* (%)	6 (20.0)	8 (22.2)	1.000
ED visit since inclusion, *n* (%)	1 (3.3)	0 (0)	0.927
GP visit since inclusion, *n* (%)	11 (36.7)	16 (44.4)	0.698
Hospitalization since inclusion, *n* (%)	1 (3.3)	0 (0)	0.927
Imaging since inclusion, *n* (%)	3 (10.0)	6 (16.7)	0.440

	Control (*N* = 26)	Intervention (*N* = 33)	
Follow-up day 21			
Loss to follow-up, *n* (%)	4 (13.3)	3 (8.3)	
Physiotherapy since last follow-up, *n* (%)	11 (36.7)	15 (41.7)	0.813
ED visit since last follow-up, *n* (%)	2 (6.7)	1 (2.8)	0.427
GP visit since last follow-up, *n* (%)	9 (30.0)	16 (44.4)	0.293
Hospitalization since last follow-up, *n* (%)	0 (0)	1 (2.8)	0.379
Imaging since last follow-up, *n* (%)	3 (10.0)	2 (5.6)	0.462

	Control (*N* = 26)	Intervention (*N* = 32)	
Follow-up day 42			
Loss to follow-up, n (%)	4 (13.3)	4 (11.1)	
Physiotherapy since last follow-up, *n* (%)	18 (60.0)	21 (58.3)	0.992
ED visit since last follow-up, *n* (%)	2 (6.7)	0 (0)	0.383
GP visit since last follow-up, *n* (%)	8 (26.7)	14 (38.9)	0.459
Hospitalization since last follow-up, *n* (%)	1 (3.3)	1 (2.8)	1.000
Imaging since last follow-up, *n* (%)	6 (20.0)	3 (8.3)	0.157

*ED* emergency department, *GP* general practitioner

**Table 5:** Adherence to therapy recommendations made at inclusion (assessed on day 7 after inclusion)

	Control (*N* = 30)	Intervention (*N* = 36)	*P* value
Adjustment of footwear, *n* (%)	1 (3.3)	10 (27.8)	0.019
Adjustment to visual aid, *n* (%)	2 (6.7)	7 (19.4)	0.026
Home-hazard intervention, *n* (%)	5 (16.7)	6 (16.7)	0.053
Regular balance exercises, *n* (%)	8 (26.7)	22 (61.1)	0.003
Regular strength exercises, *n* (%)	6 (20.0)	17 (47.2)	0.006
Regular walk, *n* (%)	16 (53.4)	29 (80.5)	0.020

## References

[1] Owens PL, Russo CA, Spector W, Mutter R (2006) Emergency Department Visits for Injurious Falls among the Elderly, 2006. Healthcare Cost and Utilization Project (HCUP) Statistical Briefs. Rockville (MD): Agency for Healthcare Research and Quality (US) 21452495

[2] Salari N, Darvishi N, Ahmadipanah M, Shohaimi S, Mohammadi M (2022) Global prevalence of falls in the older adults: a comprehensive systematic review and meta-analysis. J Orthop Surg Res 17(1):334. 10.1186/s13018-022-03222-1 35765037 10.1186/s13018-022-03222-1 PMC9238111

[3] Tinetti M et al (1988) Risk factors for falls among elderly persons living in the community. NEJM 319:1701–1707 3205267 10.1056/NEJM198812293192604

[4] Tinetti ME, Williams CS (1998) The effect of falls and fall injuries on functioning in community-dwelling older persons. J Gerontol 53A:M112–M119 10.1093/gerona/53a.2.m112 9520917

[5] Goldberg EM, Marks SJ, Resnik LJ, Long S, Mellott H, Merchant RC (2020) Can an emergency department-initiated intervention prevent subsequent falls and health care use in older adults? A randomized controlled trial. Ann Emerg Med 76(6):739–750. 10.1016/j.annemergmed.2020.07.025 32854965 10.1016/j.annemergmed.2020.07.025 PMC7686139

[6] Close J, Ellis M, Hooper R, Glucksman E, Jackson S, Swift C (1999) Prevention of falls in the elderly trial (PROFET): a randomised controlled trial. Lancet 353(9147):93–97. 10.1016/S0140-6736(98)06119-4 10023893 10.1016/S0140-6736(98)06119-4

[7] Gurley KL, Blodgett MS, Burke R, Shapiro NI, Edlow JA, Grossman SA (2020) The utility of emergency department physical therapy and case management consultation in reducing hospital admissions. J Am Coll Emerg Physicians Open 1(5):880–886. 10.1002/emp2.12075 33145536 10.1002/emp2.12075 PMC7593441

[8] Lesser A, Israni J, Kent T, Ko KJ (2018) association between physical therapy in the emergency department and emergency department revisits for older adult fallers: a nationally representative analysis. J Am Geriatr Soc 66(11):2205–2212. 10.1111/jgs.15469 30132800 10.1111/jgs.15469

[9] Hammouda N, Carpenter CR, Hung WW, Lesser A, Nyamu S, Liu S et al (2021) Moving the needle on fall prevention: A Geriatric Emergency Care Applied Research (GEAR) network scoping review and consensus statement. Acad Emerg Med 28(11):1214–1227. 10.1111/acem.14279 33977589 10.1111/acem.14279 PMC8581064

[10] Scheffer AC, Schuurmans MJ, van Dijk N, van der Hooft T, de Rooij SE (2008) Fear of falling: measurement strategy, prevalence, risk factors and consequences among older persons. Age Ageing 37(1):19–24. 10.1093/ageing/afm169 18194967 10.1093/ageing/afm169

[11] Hill K, Womer M, Russell M, Blackberry I, McGann A (2010) Fear of falling in older fallers presenting at emergency departments. J Adv Nurs 66(8):1769–1779. 10.1111/j.1365-2648.2010.05356.x 20557379 10.1111/j.1365-2648.2010.05356.x

[12] Lanoue MP, Sirois MJ, Perry JJ, Lee J, Daoust R, Worster A et al (2020) Fear of falling in community-dwelling older adults presenting to the emergency department for minor injuries: Impact on return to the ED and future falls. CJEM 22(5):692–700. 10.1017/cem.2020.383 32638689 10.1017/cem.2020.383

[13] Cumming RG, Salkeld G, Thomas M, Szonyi G (2000) Prospective study of the impact of fear of falling on activities of daily living, SF-36 scores, and nursing home admission. J Gerontol A Biol Sci Med Sci 55(5):M299-305. 10.1093/gerona/55.5.m299 10819321 10.1093/gerona/55.5.m299

[14] Li F, Fisher KJ, Harmer P, McAuley E, Wilson NL (2003) Fear of falling in elderly persons: association with falls, functional ability, and quality of life. J Gerontol B Psychol Sci Soc Sci 58(5):P283–P290. 10.1093/geronb/58.5.p283 14507935 10.1093/geronb/58.5.p283

[15] Delbaere K, Crombez G, Vanderstraeten G, Willems T, Cambier D (2004) Fear-related avoidance of activities, falls and physical frailty. A prospective community-based cohort study. Age Ageing 33(4):368–373. 10.1093/ageing/afh106 15047574 10.1093/ageing/afh106

[16] García-Martínez A, García-Rosa S, Gil-Rodrigo A, Machado VT, Pérez-Fonseca C, Nickel CH et al (2024) Prevalence and outcomes of fear of falling in older adults with falls at the emergency department: a multicentric observational study. Eur Geriatric Med. 10.1007/s41999-024-00992-1 10.1007/s41999-024-00992-1 38809489

[17] Montero-Odasso M, van der Velde N, Martin FC, Petrovic M, Tan MP, Ryg J et al (2022) World guidelines for falls prevention and management for older adults: a global initiative. Age Ageing. 10.1093/ageing/afac205 36178003 10.1093/ageing/afac205 PMC9523684

[18] Goldberg EM, Marks SJ, Ilegbusi A, Resnik L, Strauss DH, Merchant RC (2020) GAPcare: the geriatric acute and post-acute fall prevention intervention in the emergency department: preliminary data. J Am Geriatr Soc 68(1):198–206. 10.1111/jgs.16210 31621901 10.1111/jgs.16210 PMC7001768

[19] Tousignant-Laflamme Y, Beaudoin AM, Renaud AM, Lauzon S, Charest-Bosse MC, Leblanc L et al (2015) Adding physical therapy services in the emergency department to prevent immobilization syndrome—a feasibility study in a university hospital. BMC Emerg Med 15:35. 10.1186/s12873-015-0062-1 26635006 10.1186/s12873-015-0062-1 PMC4669664

[20] Schulz KF, Altman DG, Moher D (2010) CONSORT 2010 Statement: updated guidelines for reporting parallel group randomised trials. BMC Med 8:18. 10.1186/1741-7015-8-18 20334633 10.1186/1741-7015-8-18 PMC2860339

[21] World Health Organization. Step safely: strategies for preventing and managing falls across the life-course. 2021.

[22] Kellog International Work Group (1987) The prevention of falls in later life. Dan Med Bull 34:1–24 3595217

[23] Hasemann W, Grossmann FF, Stadler R, Bingisser R, Breil D, Hafner M et al (2018) Screening and detection of delirium in older ED patients: performance of the modified Confusion Assessment Method for the Emergency Department (mCAM-ED). A two-step tool. Intern Emerg Med 13(6):915–922. 10.1007/s11739-017-1781-y 29290048 10.1007/s11739-017-1781-y

[24] Nasreddine ZS, Phillips NA, Bedirian V, Charbonneau S, Whitehead V, Collin I et al (2005) The Montreal Cognitive Assessment, MoCA: a brief screening tool for mild cognitive impairment. J Am Geriatr Soc 53(4):695–699. 10.1111/j.1532-5415.2005.53221.x 15817019 10.1111/j.1532-5415.2005.53221.x

[25] Bjelland I, Dahl AA, Haug TT, Neckelmann D (2002) The validity of the Hospital Anxiety and Depression Scale. An updated literature review. J Psychosom Res 52(2):69–77. 10.1016/s0022-3999(01)00296-3 11832252 10.1016/s0022-3999(01)00296-3

[26] Zigmond AS, Snaith RP (1983) The hospital anxiety and depression scale. Acta Psychiatr Scand 67(6):361–370. 10.1111/j.1600-0447.1983.tb09716.x 6880820 10.1111/j.1600-0447.1983.tb09716.x

[27] Nickel CH, Kellett J (2023) Assessing physiologic reserve and frailty in the older emergency department patient: should the paradigm change? Clin Geriatr Med 39(4):475–489. 10.1016/j.cger.2023.05.004 37798060 10.1016/j.cger.2023.05.004

[28] Rockwood K, Song X, MacKnight C, Bergman H, Hogan DB, McDowell I et al (2005) A global clinical measure of fitness and frailty in elderly people. CMAJ 173(5):489–495. 10.1503/cmaj.050051 16129869 10.1503/cmaj.050051 PMC1188185

[29] Rueegg M, Nissen SK, Brabrand M, Kaeppeli T, Dreher T, Carpenter CR et al (2022) The clinical frailty scale predicts 1-year mortality in emergency department patients aged 65 years and older. Acad Emerg Med 29(5):572–580. 10.1111/acem.14460 35138670 10.1111/acem.14460 PMC9320818

[30] Kempen GI, Yardley L, van Haastregt JC, Zijlstra GA, Beyer N, Hauer K et al (2008) The Short FES-I: a shortened version of the falls efficacy scale-international to assess fear of falling. Age Ageing 37(1):45–50. 10.1093/ageing/afm157 18032400 10.1093/ageing/afm157

[31] Guralnik JM, Simonsick EM, Ferrucci L, Glynn RJ, Berkman LF, Blazer DG et al (1994) A short physical performance battery assessing lower extremity function: association with self-reported disability and prediction of mortality and nursing home admission. J Gerontol 49(2):M85-94. 10.1093/geronj/49.2.m85 8126356 10.1093/geronj/49.2.m85

[32] Beratungsstelle für Unfallverhütung. Selbstständig bis ins hohe Alter. Wohnen, sich bewegen, mobil bleiben. Available from: https://www.bfu.ch/de/ratgeber/zuhause-sturzsicher-einrichten. Accessed on Dec 21, 2023.

[33] Delbaere K, Close JC, Mikolaizak AS, Sachdev PS, Brodaty H, Lord SR (2010) The Falls Efficacy Scale International (FES-I). A comprehensive longitudinal validation study. Age Ageing 39(2):210–216. 10.1093/ageing/afp225 20061508 10.1093/ageing/afp225

[34] Aibar-Almazan A, Martinez-Amat A, Cruz-Diaz D, De la Torre-Cruz MJ, Jimenez-Garcia JD, Zagalaz-Anula N et al (2019) Effects of Pilates on fall risk factors in community-dwelling elderly women: a randomized, controlled trial. Eur J Sport Sci 19(10):1386–1394. 10.1080/17461391.2019.1595739 30990762 10.1080/17461391.2019.1595739

[35] Fisken AL, Waters DL, Hing WA, Steele M, Keogh JW (2015) Comparative effects of 2 aqua exercise programs on physical function, balance, and perceived quality of life in older adults with osteoarthritis. J Geriatr Phys Ther 38(1):17–27. 10.1519/JPT.0000000000000019 24743752 10.1519/JPT.0000000000000019

[36] Kapan A, Luger E, Haider S, Titze S, Schindler K, Lackinger C et al (2017) Fear of falling reduced by a lay led home-based program in frail community-dwelling older adults: a randomised controlled trial. Arch Gerontol Geriatr 68:25–32. 10.1016/j.archger.2016.08.009 27588891 10.1016/j.archger.2016.08.009

[37] Mat S, Ng CT, Tan PJ, Ramli N, Fadzli F, Rozalli FI et al (2018) Effect of modified otago exercises on postural balance, fear of falling, and fall risk in older fallers with knee osteoarthritis and impaired gait and balance: a secondary analysis. PM R 10(3):254–262. 10.1016/j.pmrj.2017.08.405 28827207 10.1016/j.pmrj.2017.08.405

[38] Begin D, Janecek M, Macedo LG, Richardson J, Wojkowski S (2022) The relationship between fear of falling and functional ability following a multi-component fall prevention program: an analysis of clinical data. Physiother Theory Pract. 10.1080/09593985.2022.2137384 36305706 10.1080/09593985.2022.2137384

[39] Scheffers-Barnhoorn MN, van Eijk M, van Haastregt JCM, Schols J, van Balen R, van Geloven N et al (2019) Effects of the FIT-HIP intervention for fear of falling after hip fracture: a cluster-randomized controlled trial in geriatric rehabilitation. J Am Med Dir Assoc 20(7):857–865. 10.1016/j.jamda.2019.03.009. (**e2**) 31078486 10.1016/j.jamda.2019.03.009

[40] Tischler L, Hobson S (2009) Fear of falling: a qualitative study among community-dwelling older adults. Phys Occup Ther Geriatr 23(4):37–53. 10.1080/J148v23n04_03

[41] Yardley L, Smith H (2002) A prospective study of the relationship between feared consequences of falling and avoidance of activity in community-living older people. Gerontologist 42(1):17–23. 10.1093/geront/42.1.17 11815695 10.1093/geront/42.1.17

[42] Delbaere K, Close JC, Brodaty H, Sachdev P, Lord SR (2010) Determinants of disparities between perceived and physiological risk of falling among elderly people: cohort study. BMJ 341:c4165. 10.1136/bmj 20724399 10.1136/bmj.c4165 PMC2930273

[43] van der Meulen E, Zijlstra GA, Ambergen T, Kempen GI (2014) Effect of fall-related concerns on physical, mental, and social function in community-dwelling older adults: a prospective cohort study. J Am Geriatr Soc 62(12):2333–2338. 10.1111/jgs.13083 25438609 10.1111/jgs.13083

[44] Kruisbrink M, Crutzen R, Kempen G, Zijlstra GAR (2021) Assessing avoidance behavior due to concerns about falling: psychometric properties of the FES-IAB in a sample of older adults of an online panel. Arch Gerontol Geriatr 97:104469. 10.1016/j.archger.2021.104469 34298258 10.1016/j.archger.2021.104469

[45] Oh-Park M, Xue X, Holtzer R, Verghese J (2011) Transient versus persistent fear of falling in community-dwelling older adults: incidence and risk factors. J Am Geriatr Soc 59(7):1225–1231. 10.1111/j.1532-5415.2011.03475.x 21718266 10.1111/j.1532-5415.2011.03475.x PMC3298667

[46] Jang SN, Cho SI, Oh SW, Lee ES, Baik HW (2007) Time since falling and fear of falling among community-dwelling elderly. Int Psychogeriatr 19(6):1072–1083. 10.1017/S1041610206004807 17288637 10.1017/S1041610206004807

[47] Scheffers-Barnhoorn MN, van Eijk M, Schols J, van Balen R, Kempen G, Achterberg WP et al (2021) Feasibility of a multicomponent cognitive behavioral intervention for fear of falling after hip fracture: process evaluation of the FIT-HIP intervention. BMC Geriatr 21(1):224. 10.1186/s12877-021-02170-5 33794804 10.1186/s12877-021-02170-5 PMC8017759

[48] Conneely M, Leahy S, O’Connor M, Corey G, Gabr A, Saleh A et al (2023) A physiotherapy-led transition to home intervention for older adults following emergency department discharge: a pilot feasibility randomised controlled trial (ED PLUS). Clin Interv Aging 18:1769–1788. 10.2147/cia.S413961 37901478 10.2147/CIA.S413961 PMC10612516

[49] Hepkema BW, Koster L, Geleijn E, E VDE, Tahir L, Oste J et al (2022) Feasibility of a new multifactorial fall prevention assessment and personalized intervention among older people recently discharged from the emergency department. PLoS One 17(6):e0268682. 10.1371/journal.pone.0268682 10.1371/journal.pone.0268682 PMC9182319 35679254

[50] Simek EM, McPhate L, Haines TP (2012) Adherence to and efficacy of home exercise programs to prevent falls: a systematic review and meta-analysis of the impact of exercise program characteristics. Prev Med 55(4):262–275. 10.1016/j.ypmed.2012.07.007 22813920 10.1016/j.ypmed.2012.07.007

[51] Carpenter CR, Malone ML (2020) Avoiding therapeutic nihilism from complex geriatric intervention “negative” trials: STRIDE lessons. J Am Geriatr Soc 68(12):2752–2756. 10.1111/jgs.16887 33079398 10.1111/jgs.16887

[52] Hoffman GJ, Ha J, Alexander NB, Langa KM, Tinetti M, Min LC (2018) Underreporting of fall injuries of older adults: implications for wellness visit fall risk screening. J Am Geriatr Soc 66(6):1195–1200. 10.1111/jgs.15360 29665016 10.1111/jgs.15360 PMC6105546

[53] Whitehead AL, Julious SA, Cooper CL, Campbell MJ (2016) Estimating the sample size for a pilot randomised trial to minimise the overall trial sample size for the external pilot and main trial for a continuous outcome variable. Stat Methods Med Res 25(3):1057–1073. 10.1177/0962280215588241 26092476 10.1177/0962280215588241 PMC4876429

